# Extreme hepatectomy with modified ALPPS in a rat model: gradual portal vein restriction associated with hepatic artery restriction

**DOI:** 10.1186/s12893-023-02197-y

**Published:** 2023-09-25

**Authors:** Xiaoqin He, Yuefeng Zhang, Peng Ma, Zuo Mou, Wei Wang, Kaihuan Yu, Weixing Wang

**Affiliations:** 1 Department of Hepatobiliary Surgery, Renmin Hospital of Wuhan University, Wuhan, 430060 China; 2 The First Clinical College, Wuhan University, Wuhan, China

**Keywords:** Hepatectomy, ALPPS, Hypertrophy, Animal model

## Abstract

**Background & aim:**

Associating liver partition and portal vein ligation (PVL) for staged hepatectomy (ALPPS) is a creative strategy for enlarging the future liver remnant (FLR) and increasing the tumor resectability rate. However, the indications for ALPPS must have a certain limit when the FLR is too small. We aimed to establish a modified ALPPS model with more widen applicability in rats.

**Methods:**

An extreme ALPPS model was established in rodents with only a 6.5% FLR. The portal vein (PV) was subjected to restriction to different degrees, then the portal vein pressure (PVP) was measured. Then, different modifications of ALPPS, including hepatic artery restriction (HAR), gradual portal vein restriction (GPVR), and GPVR-associated HAR (HAR+GPVR), were applied in the extreme ALPPS models.

**Results:**

PVL or PVR provoked an immediate increase in the PVP. The PVP in the PVR -1.28 mm, PVR -0.81 mm, PVR -0.63 mm, and PVL groups was 11.05±1.57 cmH_2_O, 16.18±1.92 cmH_2_O, 20.66±1.99 cmH_2_O, and 24.10±3.33 cmH_2_O, respectively, and the corresponding 3-day survival rate was 100%, 90.09%, 36.33% and 0, respectively. Then, in the extreme ALPPS model, the growth ratio of the FLR in the control, HAR, GPVR, and HAR+GPVR groups was 0.43±0.21, 0.50±0.16, 4.80±0.86, and 7.40±2.56, and as a consequence, the corresponding 30-day survival rate was 9.09%, 15.38%, 84.61% and 92.90%, respectively.

**Conclusion:**

ALPPS itself has a limit, and high PVP after PVL contributes to postoperative death in the extreme ALPPS model. Furthermore, a modified method for extreme ALPPS is proposed, i.e., GPVR+HAR in place of PVL, which significantly improves the survival rate of extreme hepatectomy in rat models.

**Supplementary Information:**

The online version contains supplementary material available at 10.1186/s12893-023-02197-y.

## Introduction

Associating liver partition and portal vein ligation (PVL) for staged hepatectomy (ALPPS) is an innovative surgical method that has been used in recent years to solve the problem of insufficient future liver remnant (FLR), and this technique provides an opportunity for radical surgery in some patients with advanced or massive liver cancer [1–3]. The greatest advantage of ALPPS is rapidly promoting the proliferation of the remaining liver and obtaining a safe FLR proportion [4, 5].

For some patients with multifocal bilobar colorectal liver metastasis, Schadde et al and de Santibañes M et al successfully performed monosegment ALPPS based on the Couinaud segmentation method, which only retains one liver segment±the S1 segment as the FLR [6, 7]. However, the range of applicability of ALPPS must have certain limits. When the volume of the liver lobe carrying the tumor is too large, the volume of the healthy side is too small. It can be predicted that if ALPPS is applied in such cases, the PVL procedure will affect the backflow of portal vein blood, significantly increase the portal vein pressure (PVP), and probably lead to disastrous consequences [8, 9].

Therefore, extending the applicable scope of ALPPS, which means taking less liver tissue as the FLR, is an interesting and meaningful topic. This task has not yet been studied, and we propose a solution for it: gradual portal vein restriction (GPVR) to replace PVL during the ALPPS procedure. Specifically, the PV is restricted with a sliding knot, and the thread head is kept outside the body. Then, the PV is gradually tightened step by step through this device, which avoids drastic changes in PV hemodynamics and improves the patient's tolerance. In this research, we performed liver resection (the FLR was approximately 6.5% of the total liver) with the modified ALPPS procedure, and the data preliminarily proved the feasibility and safety of the GPVR method.

## Methods

### Animals

Male Sprague‒Dawley (SD) rats, aged 8 to 10 weeks, were obtained from Hunan SJA (Changsha, China) and kept under special pathogen-free (SPF) animal conditions with constant temperature and humidity. The rats were given *ad libitum* access to standard rat chow and water. The animals were allowed to adapt to the new environment for a week before any interventions. All animals and procedures were approved by the Ethics Committee of the Animal Experiment Center of Wuhan University, all methods were carried out in accordance with relevant guidelines and regulations. This study was carried out in compliance with the ARRIVE guidelines.

### Development of the extreme ALPPS model in rats

**Fig. 1:**
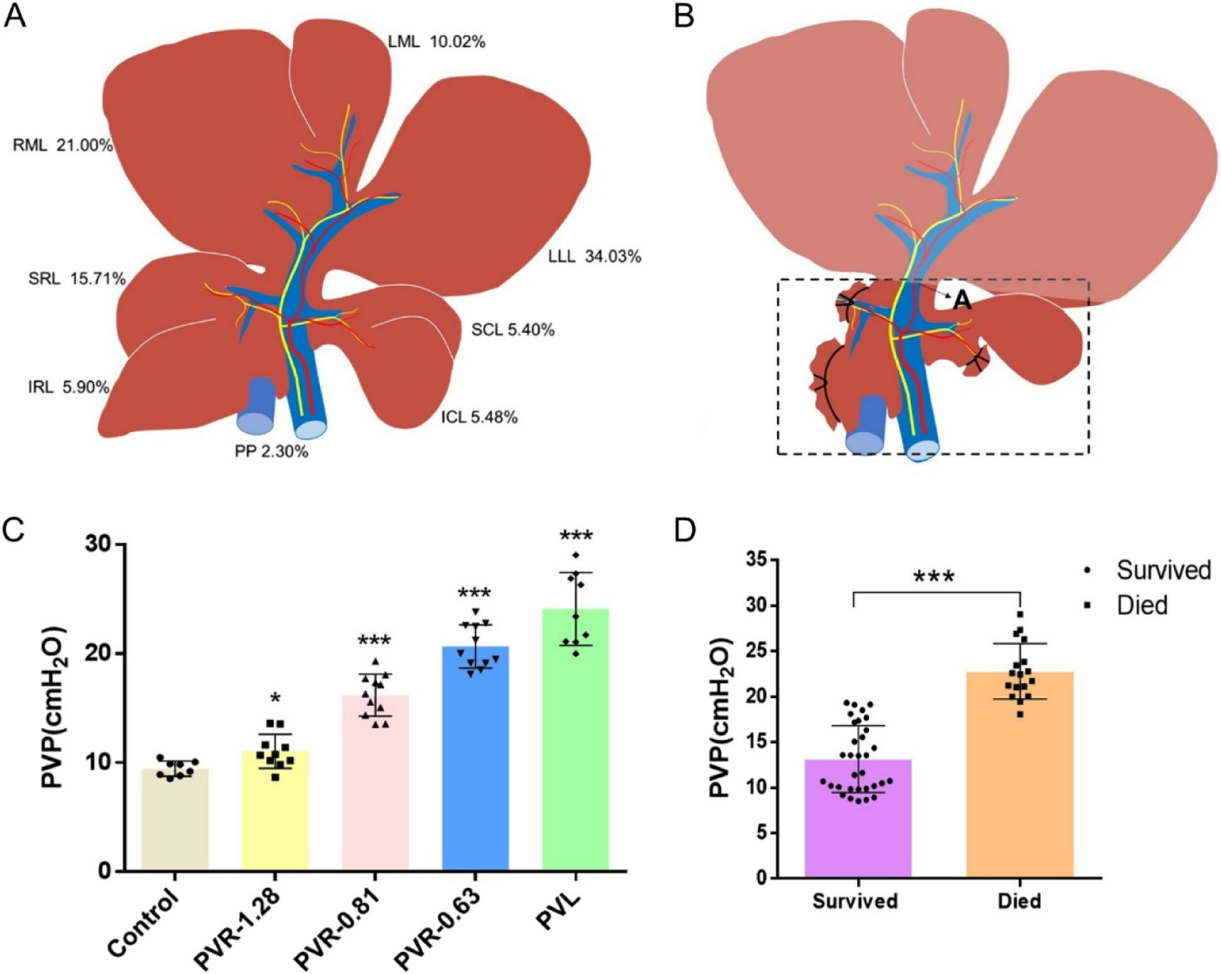
An extreme ALPPS model in rats was established, and the PVL procedure spurred the significant increase in PVP. **A** Schematic representation of SD rat liver. **B** Schematic representation of the extreme ALPPS model. SCL and part of PC were designed as the ideal future liver remnant (FLR, indicated in the dashed frame), as it represents approximately 6.5% of the total liver parenchyma. During the step I procedure, the SRL, IRL and ICL were removed, the PV was ligated or restricted at point A. The RML, LML and LLL were planned to be resected during the step III surgery. **C** The PV (at point A) was subjected to ligation or restriction at different degrees, and the portal vein pressure (PVP) was measured. (*, *P* < 0.05; **, *P* < 0.01; ***, *P* < 0.001). **D** PVPs of rats between the died and survived groups were retrospectively compared. (*, *P* < 0.05; **, *P* < 0.01; ***, *P* < 0.001). ALPPS, Associating liver partition and portal vein ligation for staged hepatectomy; FLR, future liver remnant; ICL, inferior caudate lobe; IRL, inferior right lobe; LLL, left lateral lobe; SCL, superior caudate lobe; SRL, superior right lobe; RML right medial lobe; LML left median lobe; PC pericaval tissue; PV, portal vein PVP; portal vein pressure

To verify the inference that extreme ALPPS will lead to an increase in PVP, an extreme ALPPS model was developed in rats. The superior caudate lobe (SCL) and part of the pericaval liver parenchyma (PP) were designated as the FLR (SCL+ partial PP, approximately 6.5% of the total liver) in this experiment. Since the liver lobes of rats are naturally separated from each other, the procedure for splitting the liver parenchyma is omitted. Fifty rats were randomly classified into 5 groups. The PV was thoroughly ligated or restricted at point A (Fig. 1B), and the width of the PV was measured with an electronic Vernier caliper. PVR was completed with the method described by Harvolsen and Myking using 0.63 mm (23 G), 0.81 mm (21 G), and 1.28 mm (18 G) blunt-tipped needles [10, 11]. Specifically, the PVs were ligated together with a preplaced blunt-tipped needle (21 G) lying along the PVs, and then the needle was pulled out. The degree of PVR corresponded to the thickness of the preplaced needle left behind.

### PVP measurement

After laparotomy, a 24-G cannula needle was used to pierce the mesenteric branch vein, and the tip of the cannula reached the trunk of the superior mesenteric vein. The PVP was recorded with a PowerLab 4/26 multichannel physiological recorder (AD Instruments, Australia). The average value of 3-minute measurements was regarded as the PVP.

### Laboratory tests

The bodies of recently died rats were autopsied, and these survived extreme ALPPS model rats were executed under anesthesia at fourth day after operation. The blood samples were collected and stored at -20℃. Serum assessments related to biochemical indicators, including aspartate aminotransferase (AST), alanine aminotransferase (ALT), total bilirubin (T-Bil), albumin (ALB), prothrombin time (PT), urea, creatinine (Cr) and electrolyte, were examined with a serum multiple biochemical analyzer (Siemens, Germany). The lipopolysaccharide (LPS) levels in serum were detected with a LPS enzyme linked immunosorbent assay kit.

### Experimental design

**Fig. 2:**
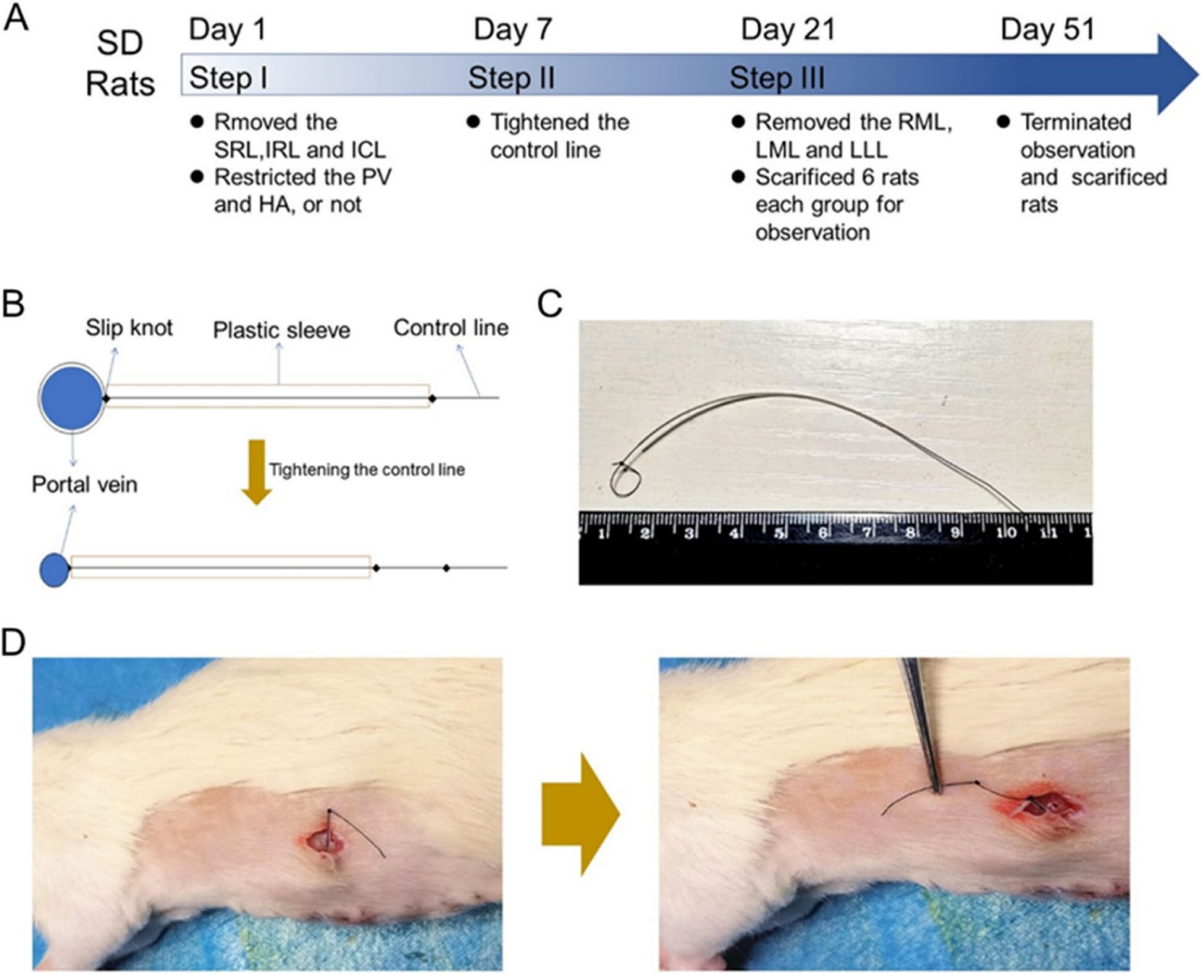
PVGR was achieved with a PV restriction device. **A** Timeline of the experimental process. **B** Schematic diagram of the PV restriction device, which is essentially a slip knot. Tightening the control line outside the body could limit the bloodstream. **C** The real picture of the PV restriction device. **D** Step II surgery. The control line was found under the skin, then tighten the control line to thoroughly block the PV, and knots were made to prevent retraction. PV, portal vein; PVGR, portal vein gradual restriction

Then, the GPVR method was tested in the extreme ALPPS models. Specifically, another 80 rats were randomly allocated to 4 groups (*n =* 20 per group): the control group, the hepatic artery restriction (HAR) group, the GPVR group, and the HAR+GPVR group. The right lobe (RL) and the inferior caudate lobe (ICL) were removed at step I in all the groups. The timeline of the experiment is presented in Fig. 2A.

### Surgical procedures

#### Step I

On day 1, anesthesia was induced or maintained by inhalation of a mixture of oxygen (0.8 L/min) and isoflurane (2.5%). The hair on the abdomen was shaved, and the skin was disinfected. Then, a 5.0-cm midline incision was made, and the liver lobes and PV were exposed. The ICL and RL were removed after ligation of the pedicles with a 4-0 silk suture. The PV and hepatic artery (HA) at point A, as shown in Fig. 1B, were separated from the Glisson sheath. For the GPVR procedure, the prepared current-limiting device (Fig. 2 B&C) was looped around the PV together with a preplaced blunt-tipped needle (21 G) lying along the PV. The current-limiting device was slowly tied, two knots were made to prevent retraction of the control line, and the needle was pulled out. The HAR procedure was performed with a similar method, and the HA (about 0.37mm) was limited to the same thickness as the 4-0 nylon silk (about 0.175mm). Finally, the incision was sutured, and the device was fixed under the skin.

#### Step II

On day 7, the current-limiting device was completely tightened to thoroughly block the blood flow of the PV. Specifically, anesthesia was conducted as before, the skin was incised, the end of the silk was found, the control line was tightened, and two knots were made to prevent retraction (Fig. 2D).

#### Step III

On day 21, 14 rats in each group were subjected to laparotomy *via* a transverse upper abdominal incision, and the liver lobes, including the left medial lobe (LML), right medial lobe (RML) and left lateral lobe (LLL), were removed. Specifically, the intraperitoneal adhesion was carefully separated, and an electric coagulation pen was used for hemostasis. The Glisson elements were found along the device and sutured, the pedicles of the LML, RML and LLL were ligated, and the lobes were excised. The abdominal cavity was closed after confirming the absence of active bleeding. After the operation, a subcutaneous injection of 5 ml of warm physiological sterile solution was administered to increase the circulatory blood volume.

### Sample collection

The animals were killed painlessly by bleeding after being placed under anesthesia on days 21 and 51. The livers were explanted with no accessory vessels or ligaments. Each individual liver lobe was weighed and recorded. Because each rat liver lobe compared to the body weight in normal healthy SD rats is constant, our previous study showed that the FLR (SCL+ part of PP)/body weight is approximately 0.00248585; thus, the FLR growth ratio was calculated using the following formula [12]:

$$Growth\;ratio=\frac{Actual\;FLR\;weight-Initial\;body\;weight\;\ast\;0.00248585}{Initial\;body\;weight\;\ast\;0.00248585}$$

### Pathological analysis

The harvested liver tissues were fixed in 4% buffered formalin for 48 hours. Standard hematoxylin-eosin (H-E) staining was conducted to observe ultrastructural changes. Immunostaining for Ki-67 was performed to evaluate hepatocyte regeneration. Sections were digitalized using a slide scanner (Hamamatsu Electronic, Japan). The number of Ki-67-positive hepatocytes was determined by manual counting in 5 random visual fields (20×).

### Statistical analysis

All quantitative data are expressed as the mean plus standard deviation (mean ± SD). The differences between multiple groups or two groups were assessed by multiple *t* test or chi-square test, respectively. The log-rank (Mantel‒Cox) test was used to analyze survival curves. The level of statistical significance was set at *P* < 0.05. Statistical analyses were performed using GraphPad Prism 6.0 software (San Diego, CA, USA).

## Results

### Portal hypertension resulted in postoperative death in the extreme ALPPS model

The extreme ALPPS rat model was established with the FLR (including the SCL and part of the PP) accounting for only approximately 6.5% of the total liver (Fig. 1A&B). The diameter of the PV (at point A) was 1.73±0.24 mm. The degree of PVR in the PVR -1.28 mm, PVR -0.81 mm, PVR -0.63 mm, and PVL groups was 45.07%, 78.00%, 86.69% and 100%, respectively. As shown in Fig. 1C, PVL or PVR provoked a significant increase in PVP in all the experimental groups. The PVP in the control, PVR -1.28 mm, PVR -0.81 mm, PVR -0.63 mm, and PVL groups was 9.47±0.67 cmH2O, 11.05±1.57 cmH_2_O, 16.18±1.92 cmH_2_O, 20.66±1.99 cmH_2_O, and 24.10±3.33 cmH_2_O, respectively. Correspondingly, PVL resulted in 100% lethality within 3 days, and the survival rate in the PVR -1.28 mm, PVR -0.81 mm, and PVR -0.63 mm groups was 100% (*P* >0.05), 90.09% (*P* =0.3806), and 36.33% (*P* =0.0045), respectively (Table 1). According to the final prognosis, these rats were divided into two groups: survived (*n=*32) and died (*n=*17) groups. PVPs between the died and survived groups were retrospectively compared, and PVP of those survived group was 13.16 ± 0.65 cmH_2_O, which was significantly lower than that of died group (22.78 ± 0.74 cmH_2_O, *P* <0.0001, Fig. 1D).

**Table 1:** The survival rates after PVR at different degrees or PVL, the results showed that excessive PVR was associated with higher mortality

**Groups**	**Survival Rates**	***P* value (chi-square test)**
Control	100% (8/8)	-
PVR-1.28	100% (10/10)	>0.05
PVR-0.81	90.90% (10/11)	0.3809
PVR-0.63	36.33% (4/11)	0.0045
PVL	0% (0/9)	< 0.0001

All deaths occurred within 3 days after operation. Chi-square test was applied for statistical analysis, and the difference was statistically significant when* P*<0.05

*PVL* Portal vein ligation, *PVR* Portal vein restriction

Based on the final prognosis, these rats were divided into two groups: survived and died groups. All these rats were sacrificed or autopsied, liver and renal function indicators as well as the LPS levels in the blood were also detected. The liver function and injury were evaluated by the serum levels of AST, ALT, T-Bil, ALB and PT; The kidney functions were evaluated with the serum levels of urea and Cr. It was observed that the mouths and ears of rats in died group were pale, while the gastrointestinal tract was dark blue due to serious hyperemia of the PV system (Figure S1). In pathological analysis, it was found that there was no significant difference in organs such as the heart, lungs, liver, and kidneys between the two groups. Compared to the survived group, the died group showed significant necrosis in the small intestinal mucosal layer and a large number of red blood cells stasis in the splenic sinus (Figure S2). What’ more, the blood LPS levels in the died group were significantly higher than those in the survived group, indicating that intestinal bacteria entered the bloodstream through the broken mucosal barrier (Table S1). In addition, the rats of died group showed more severe liver and renal dysfunction than the rats in survived group. Taken together, these results suggest that ALPPS has an indication limit and that portal hypertension induced death after extreme ALPPS.

### GPVR+HAR strongly stimulated hypertrophy of the FLR in the extreme ALPPS model

**Fig. 3:**
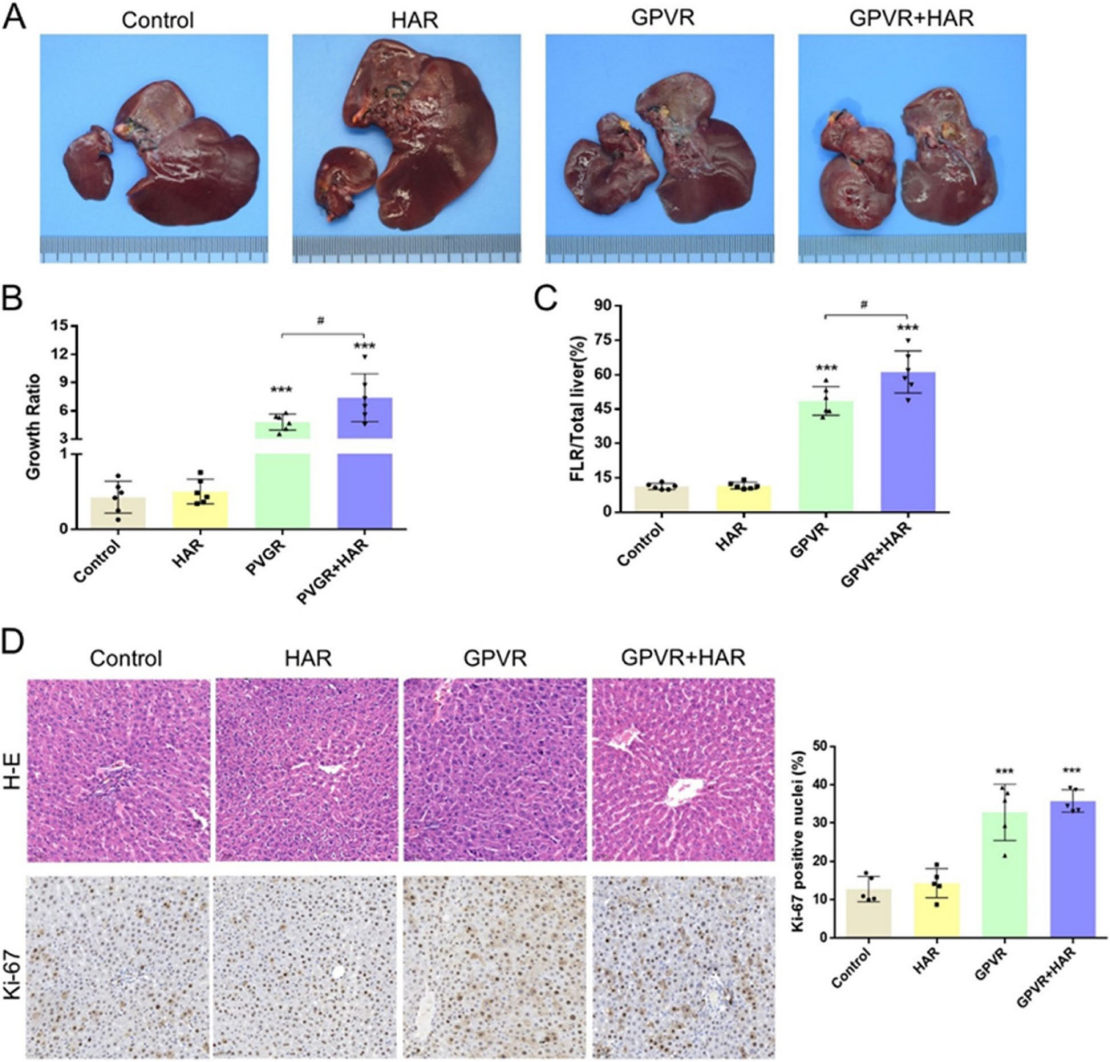
The PVGR+HAR strategy significantly triggered hypertrophy of the FLR. **A** Changes in the appearance of the whole livers at day 21. The left liver tissue was FLR (SCL+ part of PC), and the right liver parenchyma was the RML, LML and LLL. **B** The weight changes of FLR were reported with growth ratios at day 21. **C** The weight ratios of FLR to the total liver at day 21. **D** H-E and Ki-67 staining of the FLR tissues at day 21, Quantifications of Ki-67 immunostaining were also presented. (*, Compared with the control group; #, PVGR *vs.* PVGR+HAR. * or #, *P* < 0.05; ** or # #, *P* < 0.01; ***or ###, *P* < 0.001). FLR, future liver remnant; HAR, hepatic artery restriction; H-E, hematoxylin-eosin; LLL, left lateral lobe; SCL, superior caudate lobe; RML right medial lobe; LML left median lobe; PC, pericaval tissue, PV, portal vein; PVGR, portal vein gradual restriction

Next, different modifications of the ALPPS procedure were applied in the extreme ALPPS model. On day 21, the FLR growth ratio in the control, HAR, GPVR and GPVR+HAR groups was 0.43±0.21, 0.50±0.16, 4.80±0.86, and 7.40±2.56, respectively (Fig. 3A&B). Correspondingly, the FLR mass-to-whole liver weight ratio in the control, HAR, GPVR and GPVR+HAR groups was 11.21±1.33%, 11.61±1.47%, 48.53±6.22%, and 61.21±9.20%, respectively (Fig. 3C). The FLR lobe showed significant proliferation in the GPVR and GPVR+HAR groups compared to the control and HAR groups (***, *P*<0.001).

In addition, the GPVR+HAR procedure exerted a greater hypertrophic effect on the FLR lobe than the GPVR procedure (#, *P=*0.0396), and no significant difference was observed between the HAR and control groups (*P*>0.05, Fig. 3A&B).

As shown in Fig. 3D, H-E staining showed that hepatocytes were arranged tightly, and the liver trabecular structure was normal in all groups. However, the FLR in the GPVR and GPVR+HAR groups showed higher expression of Ki-67, which is consistent with the above results. Taken together, GPVR+HAR was the procedure that most strongly stimulated hypertrophy of the FLR in the extreme ALPPS model.

### Long-term survival after extreme hepatectomy with modified ALPPS

**Fig. 4:**
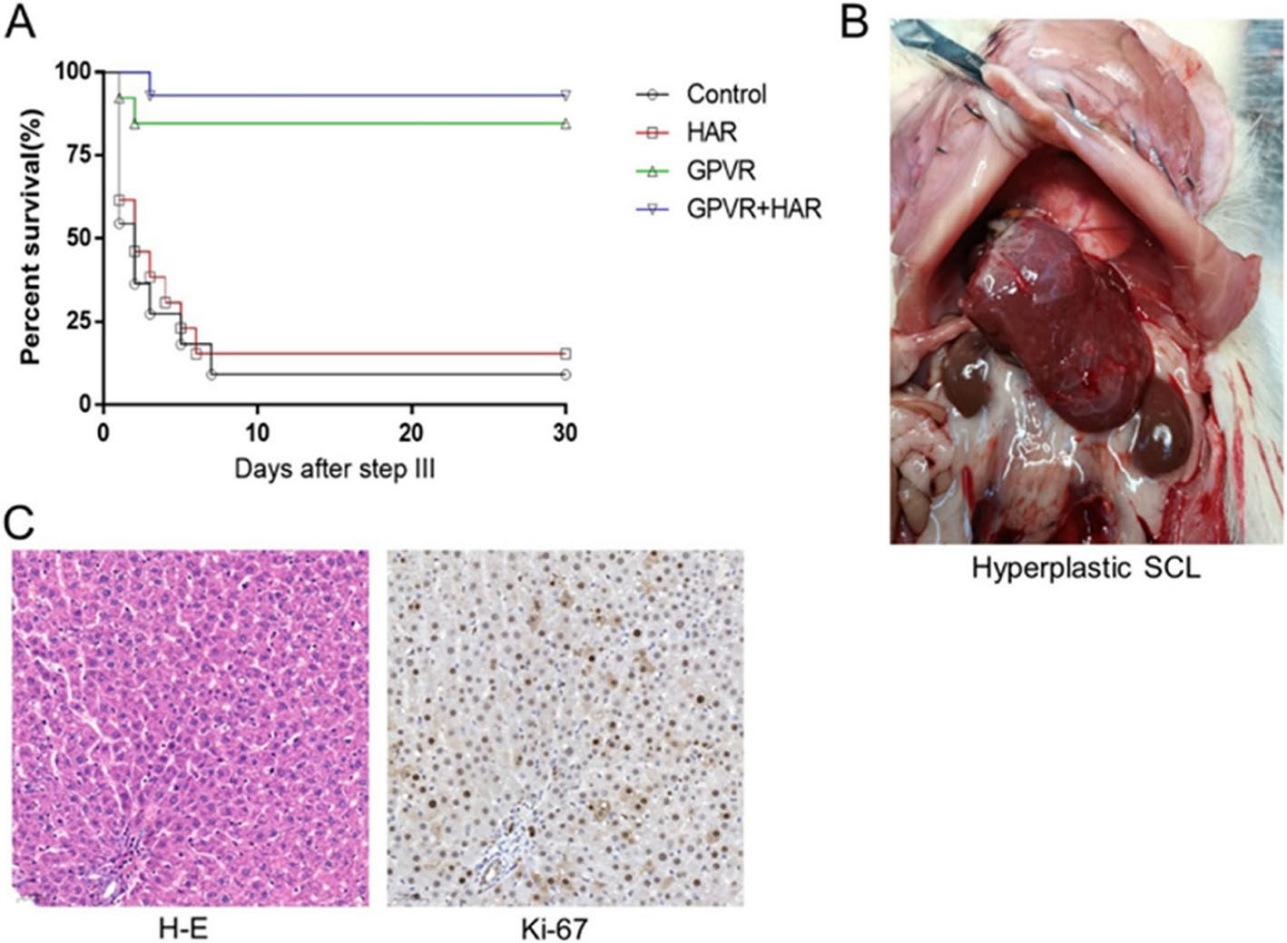
PVGR+HAR led to high survival rates of extreme ALPPS rat models. Kaplan–Meier survival curve after step III surgery. Log Rang test (Mantel-Cox) was used to analyzed survival curves. These rats achieved long-term survival with only the SCL+ part of the PC, and one of the autopsy images was presented. H-E and Ki-67 staining of FLR at day 51. H-E, hematoxylin-eosin; SCL, superior caudate lobe; PC, pericaval tissue

In each group, 14 rats were received the step III resection on day 21, the RML, LML and LLL were removed. There was a failed hepatectomy case in control and GPVR groups, respectively. As shown in Fig. 4A, almost all deaths occurred within 1 week after the step III operation. The 30-day survival rate in the control, HAR, GPVR and GPVR+HAR groups was 9.09%, 15.38% (*vs.* control, *P*=0.1772), 84.61% (*vs.* control, *P*<0.0001) and 92.90% (*vs.* control, *P*<0.0001), respectively (Table 2). Moreover, no significant difference was observed between the GPVR and GPVR+HAR groups (*P*=0.4959). These results suggest that the GPVR and GPVR+HAR surgical strategies dramatically reduced the high mortality rate in the extreme ALPPS model.

**Table 2:** The surviving rates of rats after extreme hepatectomy at day 51, and HAR+GPVR group had the highest survival rates

**Groups**	**Survival Rates**	***P* value (chi-squa1·e test)**
Control	7.70% (1/13)	-
HAR	14.29% (2/14)	0.6256
GPVR	84.61% (11/13)	< 0.0001
HAR+GPVR	92.90% (13/14)	< 0.0001

Chi-square test was applied for statistical analysis, and the difference was statistically significant when* P*<0.05

*HAR* Hepatic artery restriction, *GPVR* Gradual portal vein restriction

In addition, as presented in Fig. 4B, the FLR mass was dramatically enlarged compared to its original appearance, and the FLR mass-to-whole body weight ratio was 0.0251±0.0034, which was nearly 10 times higher than its original value. However, the liver/body weight ratio was lower than the normal level (0.0375, our previous data), and the blunt liver margin and relatively high strong Ki-67 staining indicated that the liver was still proliferating (Fig. 4B&C). These data indicate that rats could survive without complications for a long time after extreme hepatectomy with GPVR- or GPVR+HAR-ALPPS procedure, although it would take a long time for the liver to recover to the normal level.

## Discussion

ALPPS is a widely acknowledged and clinically applied two-step hepatectomy procedure [1, 2]. One prevailing theory regarding the proliferation of the FLR after ALPPS is the so-called “blood-flow” theory; PVL as well as liver splitting leads to the redistribution of PV blood, resulting in a significant increase in the volume of portal flow to the contralateral liver lobe, thus inducing proliferation of the liver on the healthy side [13–16].

Although monosegment ALPPS has been proven to be feasible in the clinic, the indications for ALPPS are certainly limited [6, 7]. When the occluded lobe is too large and the healthy side is too small, PVL will lead to hypertension of the PV system and obstruction of PV backflow.

To prove this deduction, an extreme ALPPS model was established in rats. The SCL and part of the PP were designated as the FLR (approximately 6.5% of the total liver, Fig. 1A&B). As shown in Fig. 1C, these rats were divided into four groups, and their PVs were subjected to different degrees of flow restriction or ligation. The survival rate in the control, PVR -1.28 mm, PVR -0.81 mm, PVR -0.63 mm and PVL groups was 100%, 90.09%, 36.33% and 0, respectively. Based on the final prognosis, these rats were divided into two groups: survived and died groups, and the rats of died group showed significantly higher PVP (Fig. 1D). In order to investigate the cause of death, the autopsy and pathological analysis were conducted. Liver and renal function indicators as well as the LPS levels in serum were also detected. It was found that the viscera of the died group were significantly congested (Figure S1). The pathological analysis indicated the small intestine of the died group experienced congestion necrosis (Figure S2). The elevated LPS levels in the died group implied that intestinal bacteria entered the blood through the damaged mucosal barrier (Table S1) [17]. In addition, blood biochemical tests showed that the liver and kidney functions of the died group were damaged, and electrolytes also changed, but these damages were not fatal (Table S1). The increase in urea and Cr levels may be related to a decrease in renal perfusion, which could be explained by the body's shock. In our opinion, PVL or PVR triggered complex pathophysiological processes. At first, PVL or PVR led to congestion of digestive tract and spleen, which resulted in the reduction of effective circulating blood volume and congestion necrosis of intestinal mucosa. The necrosis of intestinal mucosa would cause the displacement of gut microbiota, induced sepsis, and eventually led to shock and death.

A previous study showed that a higher degree of PVL leads to a greater increase in PVP and stimulates a stronger regenerative response from the FLR [13]. While our study improves their theory and confirms the assertion that excessively high PVP will affect blood return and cause death. Apparently, there is a limit on apply scope for the classical ALPPS method, which can’t be applied in the case that the FLR is too small. We propose a solution, i.e., GPVR to replace PVL, which could help to break through the limit on the indications for ALPPS. GPVR, restriction the PV step by step, can not only avoid excessive high PVP caused by PVL, but also stimulate FLR proliferation.

What’s more, the HA buffer response, a compensatory increase of HA flow after PVL or PV embolization (PVE), has been confirmed by previous studies [18–20]. This phenomenon means the embolized lobe can “steal” arterial blood from the remnant liver after conventional ALPPS stage-I, and this effect would be more obvious when the non-FLR side is too large [21, 22]. Aa a consequence, the HA buffer response certainly attenuate the regeneration of FLR induced by PVL or PVE [18–20]. So selective HA embolization or HA restriction (HAR) has been put up to improve the efficacy of ALPPS. The procedure for transcatheter arterial embolization-salvaged ALPPS (TAE-salvaged ALPPS) has been applied in patients with hepatocellular carcinoma and severe fibrosis/cirrhosis to achieve sufficient FLR hypertrophy after stage I of conventional ALPPS [22]. Wen Zhang et al performed hepatic artery ringing and restriction operation-associating liver partition and portal vein ligation for staged hepatectomy (HARO-ALPPS) in the treatment of giant hepatocellular carcinoma. Inspired by the above studies, it was assumed that HAR would enhance ALPPS-triggered liver proliferation [23].

In this study, the model rats were subjected to 4 groups (*n =* 20 per group): control group, HAR group, GPVR group and HAR+GPVR group. As predicted, the FLR mass-to-whole liver weight ratio on day 21 in the control, HAR, GPVR and GPVR+HAR groups was 11.21±1.33%, 11.61±1.47%, 48.53±6.22% and 61.21±9.20%, respectively (Fig. 3). Correspondingly, the 30-day survival rate was 9.09%, 15.38%, 84.61% and 92.90%, respectively. These data suggest that the GPVR+HAR method is more efficient than other procedures in boosting FLR hypertrophy (Fig. 4A). Furthermore, as shown in Fig. 4B&C, the rats achieved long-term survival after extreme hepatectomy with modified ALPPS methods (Table 2).

Currently, PVL or PVE, which suddenly and completely blocks the PV blood flow, is feasible and safe in most cases. But they cause drastic changes in hemodynamics in some extreme cases and can lead to serious consequences (Fig. 1C and Table 1). In this study, we proposed a new method and concept to process the PV, i.e., GPVR—tightening the PV with a sliding knot step by step. This programmed PVL procedure avoids provoking portal hypertension and improves the safety of step-I of extreme ALPPS. In addition, HAR procedure alone did not lead to significant change in FLR volume, but it was unexpectedly observed that HAR magnified the GPVR-triggered liver regeneration, which was in line with the previous studies [22, 23].

The GPVR+HAR strategy is a derivation and development for conventional ALPPS, and has broad prospect for clinical application. At first, the success of GPVR+HAR-ALPPS in rat models suggests that more patients with advanced liver cancer may have the chance to undergo surgical resection. However, a longer waiting time was needed to achieve an adequate FLR in the extreme ALPPS animal model, which would mean tumor progression and metastasis in patients [16]. So further clinical research on the GPVR+HAR-ALPPS is necessary.

## Conclusion

In summary, our data demonstrate that there is a limit on the indications for classical ALPPS, and high PVP induced by PVL contributes to postoperative death. Furthermore, a modified method for extreme ALPPS was proposed, i.e., GPVR+HAR in place of PVL, which significantly improves the survival rate of extreme hepatectomy in rat models. GPVR+HAR-ALPPS may broaden the apply scope of ALPPS and improve the security of ALPPS in clinical practice.

### Supplementary Information

**Additional file 1: Figure S1.** The rats of the survived and died groups were subjected to autopsy. For better observation, the dying rats were selected. Both the dying and survived rats were observed under anesthesia and oxygen inhalation. The blue arrows indicated the normal blood vessels of survived rats, the yellow arrows indicated the tortuous and dilated blood vessels of died rats. **Figure S2.** The pathological analysis on the autopsied organs. **Table S1.** Changes in liver functions between the survived and died groups. Changes in biochemical indicators and lipopolysaccharide (LPS) between survived group and died group. The liver function and injury were evaluated by the serum levels of AST, ALT, T-Bil, ALB and PT; The kidney functions were evaluated by the serum levels of urea and creatinine (Cr). *ALB, albumin; ALT, alanine aminotransferase; AST, aspartate aminotransferase; Cr, creatinine; LPS, lipopolysaccharide; PT, Prothrombin time; T-Bil, total bilirubin.*

## Data Availability

The datasets used and/or analysed during the current study are available from the corresponding author on reasonable request.

## Abbreviations

ALB: Albumin
ALPPS: Associating liver partition and portal vein ligation for staged hepatectomy
ALT: Alanine aminotransferase
AST: Aspartate aminotransferase
Cr: Creatinine
FLR: Future liver remnant
HA: Hepatic artery
HAR: Hepatic artery restriction
H-E: Hematoxylin-eosin
ICL: Inferior caudate lobe
RL: Right lobe
LLL: Left lateral lobe
LML: Left medial lobe
LPS: Lipopolysaccharide
PP: Pericaval parenchyma
PV: Portal vein
GPVR: Gradual portal vein restriction
PT: Prothrombin time
PVP: Portal vein pressure
PVR: Portal vein restriction
PVL: Portal vein ligation
RML: Right medial lobe
SCL: Superior caudate lobe
SRL: Superior right lobe
T-Bil: Total bilirubin
