# Supplementary material for: Extreme hepatectomy with modified ALPPS in a rat model: gradual portal vein restriction associated with hepatic artery restriction
Source: BMC Surg. 2023 Sep 25;23:291. doi: 10.1186/s12893-023-02197-y (PMC10521515; doi:10.1186/s12893-023-02197-y)
Supplement: Supplementary file 1 — Additional file 1: Figure S1. The rats of the survived and died groups were subjected to autopsy. For better observation, the dying rats were selected. Both the dying and survived rats were observed under anesthesia and oxygen inhalation. The blue arrows indicated the normal blood vessels of survived rats, the yellow arrows indicated the tortuous and dilated blood vessels of died rats. Figure S2. The pathological analysis on the autopsied organs. Table S1. Changes in liver functions between the survived and died groups. Changes in biochemical indicators and lipopolysaccharide (LPS) between survived group and died group. The liver function and injury were evaluated by the serum levels of AST, ALT, T-Bil, ALB and PT; The kidney functions were evaluated by the serum levels of urea and creatinine (Cr). ALB, albumin; ALT, alanine aminotransferase; AST, aspartate aminotransferase; Cr, creatinine; LPS, lipopolysaccharide; PT, Prothrombin time; T-Bil, total bilirubin. [file 12893_2023_2197_MOESM1_ESM.docx]

**Figure S1. The rats of the survived and died groups were subjected to autopsy.** For better observation, the dying rats were selected. Both the dying and survived rats were observed under anesthesia and oxygen inhalation. The blue arrows indicated the normal blood vessels of survived rats, the yellow arrows indicated the tortuous and dilated blood vessels of died rats.


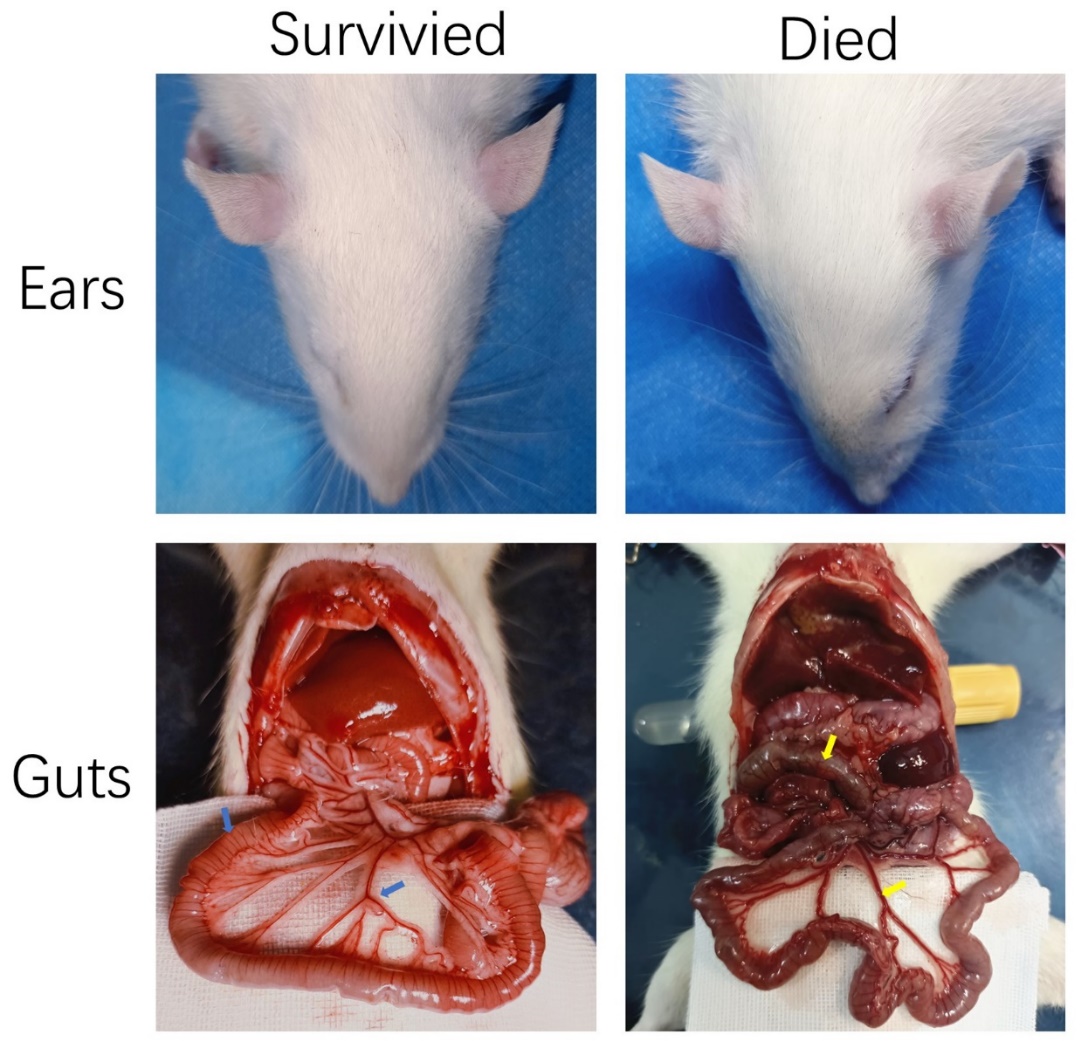


**Figure S2. The** **pathological analysis on the autopsied organs.**


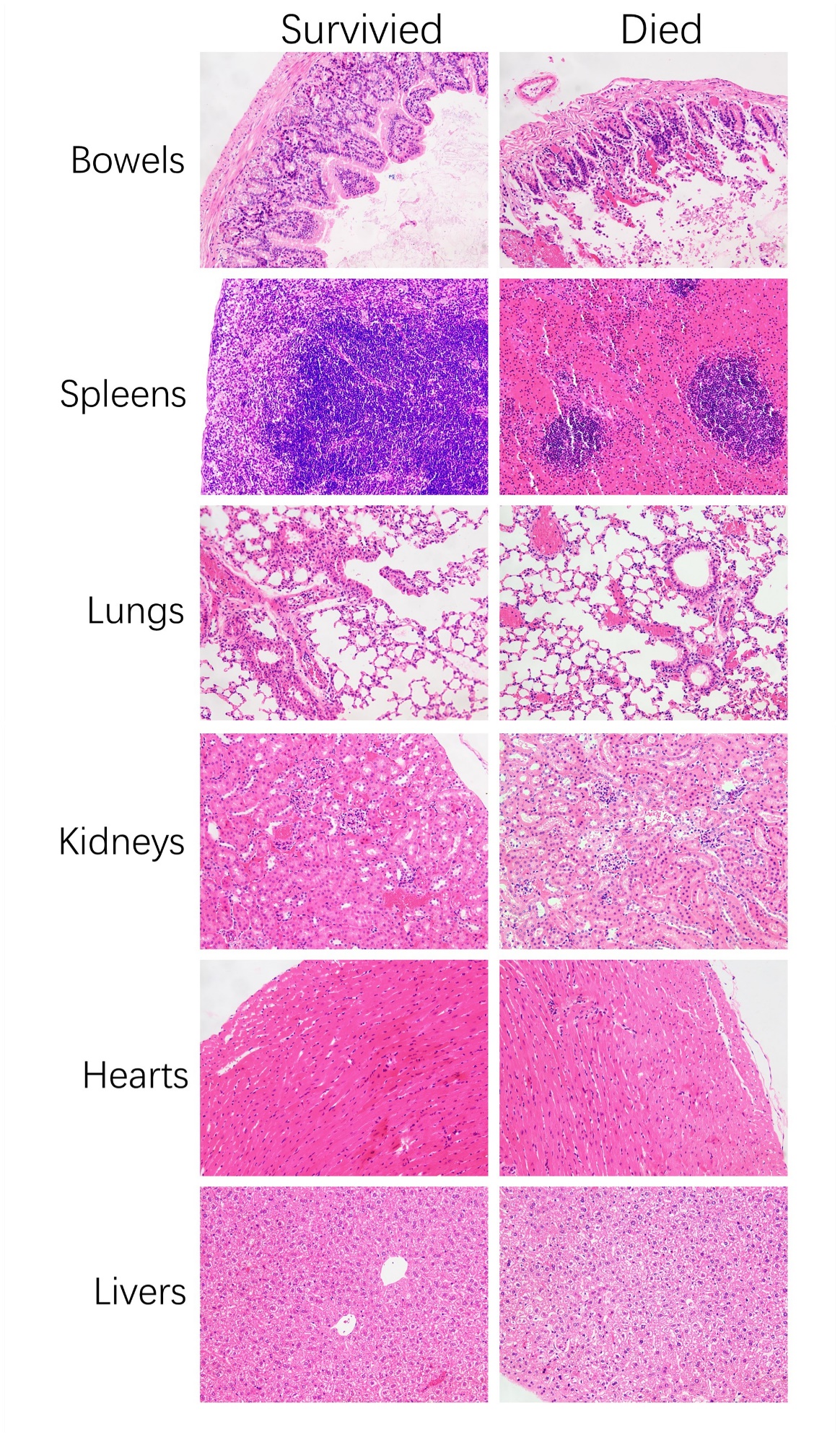


**Table S1. Changes in liver functions between the survived and died groups.** Changes in biochemical indicators and lipopolysaccharide (LPS) between survived group and died group. The liver function and injury were evaluated by the serum levels of AST, ALT, T-Bil, ALB and PT; The kidney functions were evaluated by the serum levels of urea and creatinine (Cr). *ALB, albumin; ALT, alanine aminotransferase; AST, aspartate aminotransferase; Cr, creatinine; LPS, lipopolysaccharide; PT, Prothrombin time; T-Bil, total bilirubin.*
